# Nanostructured Medical Devices: Regulatory Perspective and Current Applications

**DOI:** 10.3390/ma17081787

**Published:** 2024-04-12

**Authors:** Giuseppe D’Avenio, Carla Daniele, Mauro Grigioni

**Affiliations:** National Centre for Innovative Technologies in Public Health, Italian National Institute of Health (ISS), 00161 Rome, Italy; carla.daniele@iss.it (C.D.); mauro.grigioni@guest.iss.it (M.G.)

**Keywords:** nanomaterials, medical devices, regulatory frameworks

## Abstract

Nanomaterials (NMs) are having a huge impact in several domains, including the fabrication of medical devices (MDs). Hence, nanostructured MDs are becoming quite common; nevertheless, the associated risks must be carefully considered in order to demonstrate safety prior to their immission on the market. The biological effect of NMs requires the consideration of methodological issues since already established methods for, e.g., cytotoxicity can be subject to a loss of accuracy in the presence of certain NMs. The need for oversight of MDs containing NMs is reflected by the European Regulation 2017/745 on MDs, which states that MDs incorporating or consisting of NMs are in class III, at highest risk, unless the NM is encapsulated or bound in such a manner that the potential for its internal exposure is low or negligible (Rule 19). This study addresses the role of NMs in medical devices, highlighting the current applications and considering the regulatory requirements of such products.

## 1. Introduction

The domain of medical devices is very diversified: Actually, there are hundreds of thousands of different types of medical devices on the market, from plasters to artificial heart valves, from contact lenses to particle accelerators for cancer therapy. This multiplicity is reflected in the definition according to the Regulation (EU) 2017/745 [1]:

“‘Medical device’ means any instrument, apparatus, appliance, software, implant, reagent, material or other article intended by the manufacturer to be used, alone or in combination, for human beings for one or more of the following specific medical purposes:

- diagnosis, prevention, monitoring, prediction, prognosis, treatment or alleviation of disease,
- diagnosis, monitoring, treatment, alleviation of, or compensation for, an injury or disability,
- investigation, replacement or modification of the anatomy or of a physiological or pathological process or state,
- providing information by means of in vitro examination of specimens derived from the human body, including organ, blood and tissue donations, and which does not achieve its principal intended action by pharmacological, immunological or metabolic means, in or on the human body, but which may be assisted in its function by such means.”

The increasing diffusion of nanomaterials (NMs), i.e., materials with at least one external dimension <100 nm, is being observed also in the domain of medical devices (MDs) [2]. The peculiar characteristics of such materials (above all, the tunability of their physicochemical properties as a function of their size) are gaining much interest in the biomedical area, in light of the possibility to enhance the biocompatibility of MDs by using nanomaterials. Besides the theoretical advantages, though, the associated risks must also be carefully considered.

The objective of this study is to present the current applications of nanomaterial technology to the fabrication of MDs. The scope of this review is relative to applications that have passed the proof-of-principle test, even though they may not be necessarily close to the market. Moreover, the regulatory requirements of such products are considered.

## 2. Nanomaterials and Medical Devices

The unique properties of materials at the nanoscale (1 nm = 10^−9^ m) are often unexpected given the corresponding properties that these materials present in bulk. Consequently, the utilization of nanomaterials in medical devices is gaining success, as it can take advantage of some or all of the following properties:

- A high degree of chemical reactivity;
- A high surface-to-volume ratio, which entails a high reaction rate due to the increased surface available for reactions;
- A high potential for nanoparticle internalization and subsequent cellular responses;
- The materials’ properties at the nanoscale are affected by quantum mechanical effects, which do not apply to materials at larger scales; in the former case, size-dependent properties are also observable (e.g., emission frequency in quantum dots).

Properties such as melting point, fluorescence, electrical conductivity, magnetic permeability, and chemical reactivity change as a function of the size of the particle.

Together with these remarkable properties, it is necessary to also consider the risks associated with NMs in medical devices.

## 3. Evaluation of Nanomaterials in MDs

The Medical Device Regulation (MDR) [1] acknowledges the particular risks related to NMs, see Recital (15): “In the design and manufacture of devices, manufacturers should take special care when using nanoparticles for which there is a high or medium potential for internal exposure. Such devices should be subject to the most stringent conformity assessment procedures.”

The evaluation of materials in MDs is generally aimed at elucidating the biological effects of the device as can be found on the market. The basis of the analysis is to be found in Annex I—General Safety and Performance Requirements of the MDR. In general, “Devices shall achieve the performance intended by their manufacturer and shall be designed and manufactured in such a way that, during normal conditions of use, they are suitable for their intended purpose. They shall be safe and effective and shall not compromise the clinical condition or the safety of patients, or the safety and health of users or, where applicable, other persons, provided that any risks which may be associated with their use constitute acceptable risks when weighed against the benefits to the patient and are compatible with a high level of protection of health and safety, taking into account the generally acknowledged state of the art.”

Chapter II of Annex I considers the requirements regarding design and manufacturing, which are especially relevant for the use of nanomaterials in MDs: “Devices shall be designed and manufactured in such a way as to reduce as far as possible the risks linked to the size and the properties of particles which are or can be released into the patient’s or user’s body, unless they come into contact with intact skin only. Special attention shall be given to nanomaterials.”

Moreover, “Devices shall be designed and manufactured in such a way as to reduce as far as possible the risks posed by substances or particles, including wear debris, degradation products and processing residues, that may be released from the device.”

In general, regarding the use of materials in MDs, the following should be applied to the biological testing of medical devices according to ISO 10993-1 [3]:

“(a) The tests must be performed on the final product, or on representative samples taken from the final product or from materials processed in the same manner as the final product
(b) The choice of test procedures must take into account:
  (1) the nature, degree, duration, frequency and conditions of exposure or contact of humans with the device in normal intended use;
  (2) the chemical and physical nature of the end product;
  (3) the toxicological activity of the chemical elements or compounds in the formulation of the final product; […]
(c) If device extracts are prepared, the solvents and extraction conditions used must be appropriate to the nature and use of the final product.”

Requirement (a) reflects the fact that the standards of the ISO 10993 series have a scope that does not include—at yet—nanostructured MDs: Testing the latter, or components thereof, in order to obtain the release of nanomaterials from the device would entail unwanted size-dependent effects that must not be considered in traditional MDs. The extracted NMs may well rearrange themselves in a form not representative of what is to be found in the final product or be subject to physicochemical modifications during the process of extraction. Therefore, as discussed more broadly in subsequent Section 9, nanomaterials themselves need to be evaluated instead of extracts. It can be stated that stable methodologies for testing nanostructured MDs are not yet available and that this challenging problem still requires non-trivial research efforts.

## 4. Potential Health Effects Associated with Nanomaterials

The safety of nanomaterials has been identified as a critical problem mostly in relation to nanoparticles (NPs). The European Commission (EC) and SCENIHR (i.e., EC Scientific Committee on Emerging and Newly Identified Health Risks) published in 2015 the Final Opinion on the “Guidance on the Determination of Potential Health Effects of Nanomaterials Used in Medical Devices” [4]. The Guidance provides information on how to evaluate the risks associated with an MD fabricated with nanomaterials. The document states that “the potential risk from the use of nanomaterials in medical devices is mainly associated with the possibility for release of free nanoparticles from the device and the duration of exposure”.

Of primary importance in assessing the health effects of nanomaterials is the consideration of the latter’s toxicological potential. Nanotoxicology research is not a straightforward activity since it is still hampered by several causes: suboptimal in vitro models, lack of in vitro–in vivo correlations, variability within in vitro protocols, deficits in both material purity and physicochemical characterization [5]. This notwithstanding, many research efforts have been spent, providing many insights about NPs’ toxic effects on cells and tissues. 

Nanoparticles are able, in general, to easily cross the cellular membrane [6], which calls for an assessment of the possible nanotoxicity due to NP accumulation in cells prior to their use. Reference [7] provides evidence of the direct relationship between the incubation time and quantity of Fe_3_O_4_ NPs in mouse embryonic fibroblasts.

Several endocytic pathways are involved in the internalization of NPs. The latter’s physicochemical properties have an evident role in this process [8].

Another already observed effect of nanomaterials is their action on the integrity of the brain–blood barrier (BBB). Poly(butylcyanoacrylate) (PBCA) nanoparticles were the first polymer-based nanoparticle system used to deliver drugs to the central nervous system [9]. The influence of nanoparticles on BBB integrity was studied in reference [10] by measuring the dependence of the transendothelial electrical resistance (TEER) on NP concentration. The TEER correlates with the cell layer’s permeability and tightness. BBB integrity after treatment with PBCA nanoparticles in concentrations between 1 and 25 μg/mL over 24 h showed an increasingly marked effect of a reversible BBB loss of integrity at increasing NP concentrations until an irreversible barrier breakup occurred at the highest concentrations of NPs.

Relatively little is known about the adverse effects of nanomaterials on human health and the environment. Nanostructured MDs, in particular through the release of nanoparticles, can lead in principle to a number of adverse health effects. Of these, lung toxicity of nanoparticles shed by such devices should be considered [11]: Pulmonary toxic effects of nanoparticles and their disturbance of the pulmonary inflammatory response are being recognized as relevant problems. In vivo studies have shown that carbon-based nanoparticles, metal-based nanoparticles, oxide-based nanoparticles, and sulfide-based nanoparticles cause a pulmonary inflammatory response in mice or rats after respiratory exposure. Mesoporous carbon nanoparticles (MCNs) induce biophysical inhibition of the natural pulmonary surfactant, which increases the alveolar surface tension, thereby leading to severe alveolar collapse in mice [12]. Carbon dots (CDs) induce acute lung inflammation, and airway macrophages have been identified as target cells of CDs [13]. No more than a single exposure to graphene oxide (GO) induces lung inflammation by causing DNA damage in the lung alveolar epithelium of C57Bl/6 mice [14].

These and many other literature findings witness that the respiratory system represents a distinctive target for NP-related toxicity. This is because, aside from serving as the primary entry point for inhaled particles, it also receives the entirety of blood circulation. Consequently, there exists the possibility of lung exposure to nanoparticles not only through inhalation but also via alternative exposure pathways leading to systemic distribution, such as dermal and gastrointestinal absorption, as well as direct injection.

Not surprisingly, in the case of pre-existing pathological conditions, the risks of NMs are enhanced: There are several indications that the adverse effects of inhaled multi-walled carbon nanotubes (MWCNTs) are exacerbated whenever inflammation conditions (e.g., allergic asthma) are already present. Actually, MWCNTs are detrimental to pre-existing allergic airway inflammation in mice [15,16].

Whilst nanomaterial immunotoxicity and cytotoxicity are being actively studied, there are still limited data on the potential genotoxicity (i.e., ability to cause DNA damage) of NMs. Reference [17] presents an interlaboratory comparison study aimed at assessing five different nanobiomaterial (NBM) formulations, each with different uses. The results show that the alkaline comet assay can be suitably applied to the pre-clinical assessment of NBMs, as a reproducible and repeatable methodology for assessing NBM-induced DNA damage. 

Also, the pro-mitogenic activity of NPs should be considered, as shown in reference [18] for ZnO NPs at low concentrations. In particular, the presence of ZnO NPs was shown to stimulate pro-mitotic levels of cyclin B1.

Another potential issue arises from the unwanted impact of NPs on the coagulation system [19]. Nanoparticles can be designed to exhibit procoagulant properties or to transport coagulation-initiating factors in order to treat specific disorders. Similarly, in addressing other pathological conditions involving coagulation-related concerns, NPs can be engineered to act as anticoagulants or to carry anticoagulant drugs. Regrettably, the interface between the coagulation system and engineered nanomaterials does not consistently yield favorable outcomes: Undesired pro- and anticoagulant characteristics of nanoparticles pose significant challenges for nanomedicine applications.

Upon entry into the systemic circulation, NPs promptly interact with blood cells, proteins, endothelial cells, as well as vital components of the coagulation system, such as platelets and plasma coagulation factors. Thus, undesirable changes in the balanced function of these cells and proteins can be induced by this interaction, potentially leading to severe and life-threatening toxicities. Coagulation disorders caused by perturbation of the blood coagulation system (in this case, NP-induced coagulopathies) may manifest, for instance, in epidemiological evidence: Numerous studies have indicated that environmental NPs remarkably elevate the risk and exacerbate the prognosis of cardiovascular diseases since they can induce thrombotic complications [20,21]. Furthermore, a growing body of research points out that engineered nanomaterials may induce severe toxicity by disturbing the hemostatic balance through perturbation of the coagulation system. A common coagulation disorder, deep vein thrombosis (DVT), is characterized by clot formation in deep veins, which can be life-threatening, as dislodged clots may migrate to the lungs and trigger pulmonary embolism. Incidents of vascular thrombosis, associated with certain MNs, have been documented [22].

The interaction of NPs with plasma proteins is relevant for assessing unfavorable interactions between nanoparticles and the coagulation system, as protein binding can alter NP physicochemical properties [23], thereby influencing particle interaction with proteins [24]. The impact of nanomaterials (and biomaterials) on the coagulation system can be categorized into two main areas: interaction with plasma coagulation factors and interaction with cells (mainly platelets, epithelial cells, and monocytes). The absorption/binding of coagulation factors onto NP surfaces may lead to either: (1) inactivation of the factors or reduced availability to other components of the coagulation cascade or (2) activation of the factors upon contact. The former may result in prolongation or deficiency in coagulation reactions, while the latter may induce undesired coagulation.

Nanoparticles’ physicochemical properties (including size, charge, density of surface groups, presence of targeting moieties, surface chemistry, and composition) dictate their effects on coagulation. Nanoparticles have the capacity to interact with and influence the activity of various components of the coagulation system, such as platelets, endothelial cells, leukocytes, and plasma coagulation factors.

In summary, an in-depth physicochemical characterization of nanoparticles is warranted in order to comprehend coagulation-related toxicities and avoid unfavorable outcomes resulting from in vivo applications of engineered nanomaterials and their derivatives, such as nanostructured MDs.

## 5. Methods

The principal literature search was performed on the Web of Science Core Collection database. The intersection (with the logical operator AND) of the results pertaining to the keywords “medical device” and “nanomaterial” provided the basis for the analysis of the relevant evidence. The search with these keywords was aimed at selecting the papers which made explicit consideration of MD application(s), in short or long term, of nanomaterials.

The analysis of the results was made in light of highlighting relevant examples for each area of application, such as dentistry, orthopedics, etc., without attempting to evaluate the quality of the specific papers in a given area. Actually, the low number of the latter’s papers, reflecting the relative novelty of the field of nanostructured MDs, would have prevented us from performing successfully a finer-grained analysis. 

Not all of the papers from the basic search were found to be useful to give a picture of current nanostructured MDs, for which a proof of principle has been demonstrated. The raw data from the basic literature search have been filtered, discarding non-relevant papers. Other causes for exclusion were insufficient maturity of the application or insufficient focus on application to MDs. The core basis of the filtered literature evidence is reported in Table 1.

## 6. Fabrication of Nanostructured Medical Devices

Several techniques can be used—at least in principle—to nanostructure materials in order to use them as part of a given medical device. Without aiming at exhaustivity, on account of the breadth of the field, techniques such as electron beam lithography [39], electrospinning [40], electro-spinning/netting (ESN) [41], hierarchical nanostructuring of carbons [42] (an efficient example of which is “nanocasting” [43]), and many others can be used for the fabrication of nanostructured MDs. 

A particularly attractive category of nanostructuring techniques is related to additive manufacturing, which has been gaining increasing success and ease of use, with decreasing costs. Such techniques can deal with challenging problems, such as the fabrication of multicomponent nanostructured MDs. A possible contribution is given by Melzer et al. [31], with a methodological improvement, OPAL. The fabrication of three-dimensional (3D) structures at the microscale is critical for several applications (e.g., microrobotics). Despite recent advancements in 3D microfabrication, complex multicomponent integration with micron-sized features remains a significant hurdle. An optical positioning and linking (OPAL) platform was proposed [31] in order to craft 3D microstructures in an accurate and precise way from two types of building blocks interconnected via biochemical interactions.

A more direct application of additive manufacturing (AM) to the nanoscale fabrication of MDs is given by nanoprinting, whose recent developments leverage additive multiphoton polymerization (MPP) [44]: Wavelengths at which a polymer is transparent are used to achieve polymerization at target locations, with resolution beyond the diffraction limit. This technique has yielded feature sizes in the range of a few tens of nanometers [45].

Nanoprinting may be used, in principle, for nanostructuring the surface of medical devices, for instance, a bone implant: This type of implantable device has been shown to have different osteointegrative behaviors in the function of their surface properties [46].

**Figure 1:**
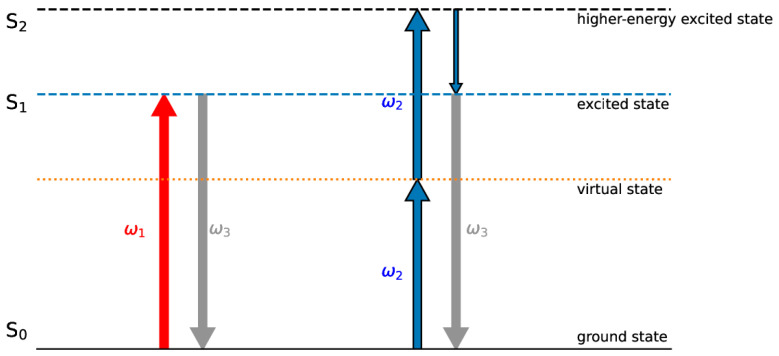
In order to induce polymerization, it is necessary to drive the photoinitiator in an excited state. On left side, one-photon absorption (OPA) is shown; on right side, the two-photon absorption (TPA) excitation process is shown.

MPP is dependent on multiphoton absorption, which requires a high photon density to drive a transition of the photoinitiator from the ground state to an excited state through a transient virtual state (refer to Figure 1). Consequently, this phenomenon is confined to the tight focal area of the laser beam, achieving nanoscale dimensions.

As shown in Figure 1, in order to induce polymerization, it is necessary to drive the photoinitiator in an excited state. On the left side, one-photon absorption (OPA) is shown, in which the excited state is reached directly with the absorption of a single photon. On the right side, the two-photon absorption (TPA) excitation process is shown: This process can take place only in a limited region, with a high photon density, enabling high printing resolutions. S_0_ is the ground state, and S_1_ is an excited state reached directly via OPA or indirectly via TPA, through a short-lived higher-energy state (S_2_) and a subsequent non-radiative decay to S_1_. Incident light frequencies are denoted as ω_1_ and ω_2_ for OPA and TPA, respectively; ω_3_ is a fluorescent emission frequency.

Due to the peculiarity of additive manufacturing (AM) fabrication methods, it is imperative to conduct thorough assessments of the biological risks for 3D-printed medical devices. An AM technique such as MPP has traditionally relied on materials such as polymers and photoinitiators: Since these have been often adapted from stereolithography that, they were not specifically formulated for biological applications. The polymerization process in MPP typically entails the generation of free-radical species, which can be potentially toxic.

Similar concerns have been noted with conventional AM techniques like Fused Deposition Modeling (FDM), where the high temperatures involved in polymer processing may present hazards. Studies have indicated that the consumable materials used in FDM printing may exhibit different chemical and physical properties post-printing, potentially leading to unintended exposure of body tissues to harmful substances leached from the printed MDs [47].

It is crucial to assess these hazards comprehensively to ensure the safety of MDs manufactured via nanoprinting and to capitalize on the capabilities afforded by this innovative technology.

## 7. Examples of Nanomaterials Used in the Fabrication of Medical Devices

The applications of nanotechnology in the field of medical devices are very numerous, especially at the research stage. In the following, some of the most promising domains of applications of nanomaterials are listed.

### 7.1. NM to Confer Antibiotic Activity

Nanomaterials have gained interest in MDs, especially for their antibacterial properties: It is well-known that implantable medical devices may be compromised by infection spreading or at least be associated with infections occurring perioperatively so that the surface properties of MDs are critical in order to curb pathogen multiplication in the body. Surgical site wound infections account for over two million nosocomial infections in patients who have been hospitalized in the United States [48].

According to a report by the Center for Disease Control, USA [49], the percentage of device-related infection varies in each application (e.g., 21% for pneumonia and 80% for urinary tract infection). The average incidence is roughly 45% of all nosocomial infections. Given the high costs associated with device-related infections, it is of the highest importance to control such infections directly at the level of MDs.

Traditionally, the prevention of device-associated infections has relied mainly on the application of good aseptic techniques in the fabrication and packaging of the MD, as well as on systemic administration of antibiotics. The efficacy of such a technique is not yet satisfactory, though [50]. Moreover, the administration of antibiotics should be avoided as much as possible in order to prevent the emergence of antimicrobial resistance. 

The application of nanotechnology to confer antibiotic (mainly antibacterial) resistance to MDs has shown that each nanomaterial has its own advantages toward specific bacteria. As remarked by Tran, N et al. [37], it is unlikely that a single nanomaterial will be found that can prevent infections caused by any type of bacteria. As a consequence, an interesting approach has been the development of combining different antibacterial nanomaterials to achieve synergic effects [51]. For instance, composite nanomaterials of silver–chitosan, silver–titanium oxide, chitosan–arginine, zinc–iron oxide, and polymer–antibiotics have shown great potential in inhibiting bacterial functions [52,53,54,55,56,57,58]. A homobifunctional imidoester-coated nanospindle (HINS) zinc oxide composite for enhancement of antibiotic efficacy is presented in reference [30]. The composite showed an antibiotic efficacy doubling that of commercialized zinc nanoparticles at the same time as having good biocompatibility, an increased surface charge, and solubility. Moreover, such composites are able to produce a large number of Zn+ ions and defensive reactive oxygen species (ROS) that effectively kill bacteria and fungi.

As with any nanomaterial in contact with the body, the cytotoxicity associated with such materials must be assessed, but in this case, this is even more compelling since most antimicrobial nanomaterials show toxicity toward one or more human cell types. 

Reference [36] discusses various nanomaterial-based approaches, such as the use of metallic and metal oxide nanoparticles and polymer-based nanocomposites, which are currently being developed for prevention and treatment of biofilms.

In reference [26], strategies were presented that combine multiple layers of defense to increase the antibacterial properties of implantable devices. In particular, a copolymer coating based on 2-hydroxypropyl acryl-amide and N-benzophenone acrylamide (a photoreactive polymer) was combined with optimally sized antimicrobial selenium nanoparticles (Se NPs). The photoreactive polymer allowed the crosslinking and covalent anchoring of the coating in a single step. The already good properties of the coating (i.e., the exceptionally low attachment of bacteria) further improved the additional bactericidal functionality associated with the incorporation of the antimicrobial Se NPs. In the cited study, moreover, an attempt has been made to modulate the release of NPs from the coatings of implantable MDs by tailoring coating parameters, such as, e.g., the nanoparticle-to-polymer ratio, in order to have controllable antibactericidal properties. This approach is not completely coherent with the cautionary approach adopted by the Medical Device Regulation (MDR) [1] about NP release in the body though (see Section 8.2). 

Peptide nanomaterials are becoming increasingly successful in, e.g., pharmaceutical research. The use of ultrashort peptides, composed of seven or fewer amino acid monomer units, with the ability to form supramolecular structures, has been reported also for their antibiotic properties. Reference [35] presents evidence about the ability of ultrashort fluorenyl-9-methoxycarbonyl (Fmoc) peptide gelators to eradicate established bacterial biofilms implicated in a variety of medical device infections (Gram-positive: *Staphylococcus aureus*, *Staphylococcus epidermidis* and Gram-negative: *Escherichia coli*, *Pseudomonas aeruginosa*). 

### 7.2. Orthopedic Applications of NM 

Several applications of nanotechnology have been proposed across many clinical domains of orthopedics. Intervertebral disc (IVD) regeneration has been often investigated with the use of cell-based therapies and NM. Injection therapy with poly (γ-glutamic acid) nanocomplexes was found to enhance the recovery of the native IVD matrix [59,60]. 

Alongside disc regeneration, nanotechnology might enable easier spinal fusion and circumvent the expenses and potential risks linked with recombinant human bone morphogenetic protein (rhBMP). Alterations to the surface of titanium spinal implants utilizing titanium oxide and zirconia NPs have exhibited the potential to enhance bone formation and reduce resorption with respect to traditional smooth implants [61].

With regard to oncologic patients, they can often undergo bone cancer resections to receive orthopedic implants. When the latter are made with standard materials, however, they lack the property of inhibiting the growth or recurrence of cancer. Among the promising implants designed to encourage normal bone growth while preventing cancer growth, nano-selenium implants have been demonstrated to inhibit the growth of malignant osteoblasts while promoting healthy bone function at the implant–tissue interface [62]. Unlike untreated titanium implants, the selenium nanomaterial increased bone adhesion, calcium deposition, bone proliferation, and alkaline phosphatase activity.

Primary joint replacement surgery is a highly successful field of application of MDs, but a serious shortcoming is given by their limited longevity. Thus, arthroplasty research is being conducted with the objective of developing implantable with a longer expected lifespan by means of tailoring specific surface characteristics of the implant, thereby achieving a more favorable interaction between the implant and the native bone. Nanotextured implant surfaces have augmented the function and growth of osteoblasts to increase implant osseointegration [63].

Improvement of the biocompatibility and mechanical properties of titanium implants [64] have been obtained using the technique of severe plastic deformation (SPD), which breaks down the coarse grains of metals into the nanoscale range by exposing the metal to a complex high-stress state.

Apart from NM-enabled surface modifications, volume effects of NMs have also been proposed in arthroplasty devices: ultra-high molecular weight polyethylene (UHMWPE) implants have been regarded with interest due to their favorable biocompatibility properties and wear resistance, but their success has been limited by the concern for potential fracture. The addition of carbon nanotubes to this material to create a novel composite has demonstrated translational success and may eventually have utility as an acetabular lining or tibial component [65].

Titanium and its alloys are often used in orthopedic surgery and dentistry due to their excellent corrosion resistance, high mechanical strength, and low density. In order to improve their biocompatibility, hydroxyapatite (HAP) is often coated on the surface of metallic implants by plasma spraying. NPs can be used to coat titanium-alloy-based implantable MDs for orthopedic applications. A study [38] presented the comparison of HAP coating vs. n-HAP (nano HAP) coating: The sintered n-HAP coated Ti-6Al-4V possessed a nobler open circuit potential but a higher corrosion current density (which reflects the corrosion rate) due to the presence of micro-cracks and pores in the coating.

### 7.3. Nanocomposites for Dentistry Applications

Nanocomposites are multiphase solid materials where one of the phases has at least one dimension of less than 100 nm, or alternatively, the structures have characteristic repeat distances at the nanometer scale between the different phases that make up the material.

These innovative materials have physical properties that can be finely tuned by changing the dimensions of the nanoscale component. Typically, a nanocomposite is composed of a bulk matrix and a nanoscale component. The mechanical, electrical, thermal, optical, electrochemical, and catalytic properties of the nanocomposite will differ remarkably from those of the component materials.

The mechanical behavior of nanocomposites must be attributed to the very high surface/volume ratio of the reinforcing phase. Also, the presence of a high aspect ratio of the latter, if the case, is relevant. The reinforcing material is given by nanoparticles, sheets (e.g., graphene sheets), or fibers. The area of the interface between the matrix and reinforcement phase(s) is much greater in nanocomposites than in conventional composite materials: Typically, there is an order of magnitude difference between the two cases. The local material properties in the nanocomposite vary considerably from the bulk of the matrix to the zone in the vicinity of the reinforcement phase(s) [66].

In these materials, it is often found that a relatively small amount of the nanoscale reinforcement has a macroscopic effect on the properties of the resulting composites, due to the large surface area of the reinforcement phase.

In general, the percentage by weight of the nanoparticulates introduced can be quite low (on the order of 0.5% to 5%), especially for non-spherical, high-aspect ratio fillers (e.g., nanosheets or nanometer-diameter cylinders, such as carbon nanotubes).

Nanocomposites have met increasing favor in healthcare, especially in dentistry. Dental composites typically contain high amounts (up to 60 vol.%) of nanosized filler particles. Several nanomaterials can be employed: silica nanoparticles [67], nanosized bioactive glasses [68,69], large ionic-density nanogel particles [70], synthetic hydroxyapatite nanocrystals, in particular when doped with F^−^ anions [71].

It may be surmised that dental personnel (and patients) may in principle inhale nanosized dust particles (<100 nm) during abrasive procedures to shape, finish, or remove restorations. In reference [72], composite dust was analyzed in real work conditions. Exposure measurements of dust in a dental clinic revealed high peak concentrations of nanoparticles in the breathing zone of both the dentist and the patient. All tested composites released very high concentrations (>10^6^ cm^−3^) of airborne particles in the nanorange, with a median diameter of airborne composite dust between 38 and 70 nm. It was found that the dust particles consisted of filler particles resin or both. The risks associated with airborne dust need to be investigated in depth, especially as far as the occupational risks are concerned due to cumulative exposure.

### 7.4. NPs for Image-Guided Cancer Therapy

The preparation of magnetic nanomaterials with useful properties is not a trivial issue. Such NMs can deliver heat, which can be a potent anticancer therapeutic agent. Generally, iron oxide nanoparticles (IONPs) are used for this purpose. The heating efficiency can be defined as the thermal power per unit mass dissipated by the magnetic material or specific loss power (SLP). The desired properties of magnetic nanomaterials in image-guided cancer therapy are high SLP and high imaging sensitivity with good spatial resolution. Currently, commercial nanoparticles do not sufficiently provide such multifunctionality. In reference [32], the preparation was reported for nanoparticles with combined functionalities, enabling both high performance for hyperthermia and imaging functionality for diagnostic and therapeutic processes.

### 7.5. Photothermal Therapy

For these applications, iron compounds in nanoparticle form have been extensively investigated. Optimal properties for these applications are photothermal conversion efficiency (PCE), blood circulation time, biostability, and cellular uptake efficiency.

Two-dimensional nanomaterials were introduced in 2004, with the landmark discovery of graphene, i.e., a nanosheet [73]. This type of NMs is very interesting and has seen different applications due to their high anisotropy and versatile chemical functions besides the high surface-to-volume ratio that is typical of all NMs.

A two-dimensional (2D) nanomaterial hybrid with metal nanoferrite nanoparticles was seen to enhance their catalytic activity and photothermal therapy (PTT) through its synergistic coupling effects [27]. This study presented a new class of cerium ferrite (CeFe2O4) nanoparticles (NPs) decorated onto branched polyethyleneimine (bPEI)-coated flower-like molybdenum disulfide nanoflowers (MoS2 NFs). This nanomaterial exhibited 53.6% PCE, which effectively induced up to 80% cytotoxicity and higher levels of ROS (reactive oxygen species) generation when exposed to the 808 nm NIR laser light (1.5 W/cm^2^, 5 min). The combination of CeFe(2)O(4) NPs with MoS2 NFs was thus found to exhibit dual-performance potential for cancer therapies (PTT and ROS generation), possibly constituting a promising new cancer therapeutic platform.

For PTT applications, but not limited to these, upconversion (UC) nanomaterials have also been proposed: These NMs have interesting properties, such as biocompatibility, near-infrared (NIR) to visible conversion, photostability, controllable emission bands, which give them the possibility to allow for versatile light delivery for deep tissue biophotonic applications. UC nanomaterials are classified into two groups: (1) lanthanide-doped UC nanoparticles and (2) triplet–triplet annihilation UC (TTAUC) nanomaterials, which are inorganic and organic, respectively. Several applications beyond PTT have also been investigated, such as photodynamic therapy (PDT), photo-triggered chemo and gene therapy, multimodal immunotherapy, NIR-mediated neuromodulations, and photochemical tissue bonding (PTB) [28].

### 7.6. Bioelectronic Interfaces

Soft bioelectronic interfaces can offer great advantages for mapping and modulating excitable networks, thereby enabling innovative MDs for monitoring and treating neural systems’ pathologies. Technological limits pose great difficulties in attaining the required resolution and coverage, though. Transition metal carbides, nitrides, and carbonitrides (MXenes) have emerged as a new class of two-dimensional (2D) nanomaterials that enable low-cost, additive-free, solution processing and can produce biocompatible films with metallic conductivity. The hydrophilic nature of MXenes enables a wide range of safe, high-throughput, and scalable processing methods using simple water-based inks (e.g., [74]). Reference [29] introduces MXtrodes, a class of soft, high-resolution, large-scale bioelectronic interfaces enabled by Ti3C2 MXene (a two-dimensional transition metal carbide nanomaterial) and scalable solution processing. The electrochemical properties of such electrodes were seen to overcome those of conventional materials; moreover, MXtrodes do not require conductive gels when used in epidermal electronics. The remarkable processability of MXenes promises to offer a rapid, low-cost, and highly scalable method for fabricating multichannel electrode arrays of arbitrary size and geometry.

### 7.7. Drug/Protein Delivery Systems

Anticancer drugs have in general low water solubility, resulting in difficult delivery to the intended tissue. However, encapsulating or entrapping these drugs within nanocarriers aids in their transportation within the bloodstream to the cancerous site: Rapid biodegradation is hampered, and drug availability is enhanced [75,76]. Moreover, nanocarriers show an enhanced permeability and retention effect (EPR) as they accumulate in cancerous tissues characterized by leaky vasculature [77]. 18β-glycyrrhetinic acid (GA) is a pentacyclic triterpene with promising hepatoprotective and anti-hepatocellular carcinoma effects. In reference [78], GA was encapsulated in core–shell NPs in order to overcome its low water solubility, which lowers its biodistribution and bioavailability, limiting its applications in biomedicine. The core–shell NPs were made of PolyD-L-lactide-co-glycolide (PLGA) coated with chitosan (CS), prepared through an osmosis-based methodology, to efficiently entrap GA. Marked differences in the cytotoxicity of the free-form and encapsulated GA were observed at the same dosage by means of real-time bioimpedance monitoring, showing that carrier-based delivery can significantly alter the concentration of the drug within the cells [78].

The comparison of biomaterials in bulk and in nanoform has been addressed directly in reference [33], considering poly-epsilon-caprolactone (PCL), i.e., a biodegradable polyester. Bulk PCL has received FDA (Food and Drug Administration, USA) and CE approval as a medical device; nevertheless, the lack of toxicity exhibited by the polymer cannot be extrapolated to its nanoform equivalent. Despite the large popularity of PCL-based NPs in the biomedical field, little data describe PCL NPs’ toxicity, particularly immunotoxicity. In reference [33], different PCL-based protein delivery systems were investigated for their immunotoxicity and hemocompatibility. Two different molecular-weight PCL polymers were used, as well as blends with chitosan and glucan. The production of PCL2 NPs and PCL2/glucan NPs was seen to be associated with the presence of NaOH, thereby causing PCL alkali hydrolysis, and generating more reactive groups (carboxyl and hydroxyl) that contributed to an increased toxicity of the NPs. The cited study points out that generalizations among different PCL NP delivery systems must be avoided, and immunotoxicity assessments should be performed in the early stage of product development. More generally, the lack of toxicity exhibited by the polymer cannot be extrapolated to its nanomaterial conformation.

### 7.8. Nano-Imaging Agents

For these products, the correct classification is far from being a trivial problem. Reference [25] summarized the hurdles encountered during the individuation of the regulatory pathway for a nano-imaging agent to track therapeutic cells during the development of cell therapy. Neither the definition of the medicinal product nor the definition of the medical device was straightforwardly applicable to the agent, which entailed continuous collaboration and communication with the relevant authorities throughout the development of the product. A more clearly defined regulatory framework for this type of nanomaterial application is necessary.

### 7.9. Medical Devices for Wound Healing

Skin lesions and burns can be difficult to heal. Nanomaterials can be combined with specific oligomers that enhance the functions of inflammatory and repairing cells, thereby enabling the fabrication of innovative MDs for accelerating wound healing. In reference [34], a natural nanotubular clay mineral (HNTs, Halloysite Nano Tubes) with good biocompatibility was combined with chitosan oligosaccharides (homo- or heterooligomers of N-acetylglucosamine and D-glucosamine): The resulting nanocomposite can be used as pour powder to enhance healing in the treatment of chronic wounds. The HTNs/chitosan oligosaccharide nanocomposite showed good in vitro biocompatibility with normal human dermal fibroblasts and enhanced in vitro fibroblast motility, promoting both proliferation and migration. The HTNs/chitosan oligosaccharide nanocomposite and the two components separately were tested for healing capacity in a murine (rat) model. HTNs/chitosan oligosaccharides allowed better skin reepithelization and reorganization than HNTs or chitosan oligosaccharide separately. The results suggest that the nanocomposite may be developed as a medical device for wound healing. The nanocomposite has also attractive production features in that it is self-assembled and formed by spontaneous ionic interaction.

In summary, the unprecedented capabilities of NMs for innovative MD fabrication have also been recognized as being associated with unforeseen hazards. It is clearly necessary to provide a suitable regulatory framework to leverage these innovative devices without undermining the safety of the users and the patients.

## 8. Regulation of Nanostructured Medical Devices in Europe

The current framework for medical devices in Europe is given by two regulations: the MDR and, for in vitro diagnostic MDs, the IVDR. The MDR repealed the previous regulatory instrument, the MDD (i.e., MD Directive 93/42/EEC), on 26 May 2021. Due to the persistence in the market of MDs regulated according to the MDD, it is interesting to consider the requirements of both regulations (MDD and MDR).

### 8.1. Medical Device Directive

Since 1993, the Medical Device Directive 93/42/CEE, together with the Directive 90/385/CEE (aimed at regulating specifically the Active Implantable Medical Devices, AIMD) has been the reference regulatory framework for MDs in Europe for almost 30 years. Both the MD and AIMD Directives were updated for the last time in 2007, with the Directive 2007/47/CE. It is to be remarked that no mention of “nanomaterial” or “nanostructure” was made therein; of course, the same applied to the previous versions of the MD Directive.

The key issue of conformity assessment for a specific medical device lies in the demonstration that the “essential requirements” of the Directive are met, thereby ensuring the safety and performance of the device. Typically, except for low-risk Class I devices, a third-party evaluation is required. The evaluation is conducted by a Notified Body, which is an accredited body independent of both the manufacturer (or its representative) and the Competent Authority for MDs.

The essential steps for evaluating the conformity of a medical device to the essential requirements typically involve a risk management phase (often in accordance with the harmonized standard ISO 14971 [79]), in vitro assessment, in vivo preclinical studies, and finally a clinical evaluation. This route typically gives the manufacturer enough information to compile the dossier of the medical device, ultimately leading to the acquisition of the CE Mark (after approval by the selected Notified Body for devices of a risk class higher than the lowest one).

### 8.2. Medical Device Regulation

On 5 April 2017, two new Regulations on medical devices were adopted, in light of replacing the MD Directives.

In particular, Regulation (EU) 2017/745 [1] replaced both the MD and AIMD Directives, and Regulation (EU) 2017/746 [80] replaced the IVD Directive 98/79/EC. The new rules were not immediately applicable: Originally, it was foreseen that the entry into force for the Regulation on medical devices was due in spring 2020, whereas for the Regulation on in vitro diagnostic medical devices, the entry into force was due in spring 2022.

Due to the COVID-19 pandemic and the resultant strain on national health authorities and medical device manufacturers, the Commission proposed postponing the implementation of the MDR by one year. This delay aimed to prevent potential shortages or delays in acquiring essential medical devices necessary for combating COVID-19. The proposal, initially put forth by the EC and endorsed by the Parliament [81], was officially published in the Official Journal of the European Union on 24 April 2020 as Regulation (EU) 2020/561 [82].

The MDR 2017/745 imposes stricter surveillance measures on the manufacturing of medical devices compared to the MDD, requiring higher levels of evidence to demonstrate conformity to the General requirements, which replace the Essential requirements of the MDD. Additionally, the MDR assigns manufacturers more stringent responsibilities regarding surveillance and reporting, as outlined in Article 88, specifically concerning Trend reporting.

Significantly, the MDR reflects the necessity for increased oversight of medical devices containing nanomaterials. According to the new regulation, medical devices incorporating or composed of nanomaterials are now categorized under Class III, the highest risk class, if there is a high or medium potential for internal exposure (Rule 19). Exceptions may apply in certain circumstances:

- Class IIb if the MD presents a low potential for internal exposure;
- Class IIa if the MD presents a negligible potential for internal exposure.

The MDR proposes the definition for nanomaterials based on Commission Recommendation 2011/696/EU [83], “with the necessary flexibility to adapt that definition to scientific and technical progress and subsequent regulatory development at Union and international level” [83]. According to Art. 2 of the MDR, the following definitions apply:

“(18) ‘nanomaterial’ means a natural, incidental or manufactured material containing particles in an unbound state or as an aggregate or as an agglomerate and where, for 50% or more of the particles in the number size distribution, one or more external dimensions is in the size range 1–100 nm; Fullerenes, graphene flakes and single-wall carbon nanotubes with one or more external dimensions below 1 nm shall also be deemed to be nanomaterials;

(19) ‘particle’, for the purposes of the definition of nanomaterial in point (18), means a minute piece of matter with defined physical boundaries;

(20) ‘agglomerate’, for the purposes of the definition of nanomaterial in point (18), means a collection of weakly bound particles or aggregates where the resulting external surface area is similar to the sum of the surface areas of the individual components;

(21) ‘aggregate’, for the purposes of the definition of nanomaterial in point (18), means a particle comprising of strongly bound or fused particles.”

As previously stated, the latest regulation designates a nanostructured medical device to the highest risk class category if there is a high or medium potential for internal exposure. This approach diverges significantly from the previous regulation (MDD), which lacked any stipulations regarding MDs containing nanomaterials. Consequently, manufacturers must provide documentation that was not explicitly mandated by the preceding regulatory framework to demonstrate compliance with the Medical Device Regulation (MDR). Consequently, there is a need for guidance to be provided to both manufacturers and stakeholders.

## 9. Normative Situation with Respect to Nanostructured Medical Devices

Standards are voluntary technical specifications that apply to various products, materials, services, and processes. They can aid in cost reduction, safety enhancement, competition improvement; moreover, they can facilitate and innovation acceptance [84]. Traditionally, ISO 10993 series standards (e.g., [3]) address the biocompatibility and toxicity of biomaterials, but the scope of these standards does not foresee the use of nanostructured materials. The significance of ISO 10993 standards lies in their recognition as harmonized standards within the MDD framework [85]. Compliance with these standards, published in the Official Journal of the European Union, implies conformity with the Essential requirements of the MDD (as per Article 3). The importance of harmonized standards has also been highlighted by the European Commission in the context of the new MD Regulation. Article 8 stipulates that “Devices that are in conformity with the relevant harmonised standards, or the relevant parts of those standards, the references of which have been published in the Official Journal of the European Union, shall be presumed to be in conformity with the requirements of this Regulation covered by those standards or parts thereof.”

However, it is currently impossible to adopt harmonized standards referring to the MDD to presume conformity with the General requirements—safety and performance (GSPR) of the MDR. Article 3 of Implementing Decision (EU) 2020/437 [86] confirms this point:

“Harmonised standards for medical devices developed in support of Directive 93/42/EEC and set out in Annexes I and II to this Decision may not be used to confer a presumption of conformity with the requirements of Regulation (EU) 2017/745.”

The Commission and the Competent Authorities are working together with the European Standardization Organizations to finalize the standardization request related to the MDR, which is required for harmonization. As of the time of writing, there are just 18 published harmonized standards supporting the MDR [86,87].

Considering the paucity of harmonized standards for the MDR, it is nevertheless natural for manufacturers to refer also, if necessary, to the harmonized standards associated with the previous regulation (MDD). This is coherent with the principles expressed by the International Medical Device Regulators Forum (IMDRF): “[Standards] represent the consensus of a variety of experts and interested entities, and a commitment to their use presents an opportunity to promote the global harmonisation of regulatory processes. Regulatory Authorities (RA) and all interested stakeholders should support and contribute to standards development to encourage the publication of standards that are useful in the regulation of medical devices and can streamline review processes” [88].

Thus, the availability of international standards, such as the harmonized standards supporting the MDD, signifies an established consensus among experts in the field of MDs. These standards could serve as a foundation to demonstrate compliance with the new MD Regulation 2017/745 while considering Article 3 of the Implementing Decision (EU) 2020/437 [86].

The ISO 10993-1 standard [3] represents a combination/harmonization of several international and national standards and guidelines related to the biological evaluation of medical devices. It is designed to offer general guidance for selecting tests to assess biological responses pertinent to the safety of medical devices and materials. Additional standards in the 10993 series cover specific aspects of biological evaluation for MDs, including cytotoxicity, sensitization, irritation, intracutaneous reactivity, systemic toxicity (acute toxicity), subacute and subchronic toxicity, genotoxicity, local effects of implantation, and hemocompatibility.

In addition to these assessments, specific MDs may necessitate supplementary investigations such as chronic toxicity, carcinogenicity, reproductive and developmental toxicity, and biodegradability. The relevance of ISO 10993 standards also for MDs containing nanomaterials is backed by document [4], which discusses the safety evaluation of nanomaterials in medical devices within the general framework outlined in ISO 10993-1:2018 [3].

Despite the continuing relevance of ISO 10993 standards, it is worth noting that only some of them have been recognized as harmonized standards for the MDR [87]. However, for the domain of nanostructured MDs, there are challenges that cast doubt on the applicability of ISO 10993 standards to such products.

To address these challenges, the ISO published a technical report in 2017 providing guidance for evaluating nanomaterials in MDs [89]. This document outlines a general approach to the biological evaluation of nanomaterials in the context of medical device evaluation and discusses how other parts of the ISO 10993 series can be utilized when evaluating nanomaterials.

The report highlights that many traditional tests used for evaluating MD biocompatibility may fail in the presence of nano-objects due to interactions of the latter with dyes used in assays such as MTT, XTT, lactate dehydrogenase, and dichlorofluorescin [89].

For example, regarding cytotoxicity testing according to ISO 10993-5 [90], reference [91] provides evidence of photocatalytic interactions between titanium dioxide (TiO_2_, titania) nanoparticles and the MTT cytotoxicity indicator. These interactions lead to the reduction of MTT and the formation of purple formazan under biologically relevant conditions. The precipitation of formazan was observed to be proportional to the concentration of TiO_2_; in particular, under laboratory daylight exposure this effect was found to be enhanced. Up to 14% false increases in viability, induced by the NP-dye reaction, were found, leading to a potential underestimation of the toxicity.

Conversely, other findings suggest an opposite effect: Data from A549 cells incubated with carbon nanotubes showed a significant cytotoxic effect within the MTT assay after 24 h, while no cytotoxicity was detected with WST-1 [92].

In light of such contrasting results, it is difficult to disagree with this indication: “corroboration of several test results from different methodologies might be required for a scientifically sound interpretation” [89].

A remarkable recommendation for designing a test plan is that “In general, nanomaterials themselves need to be evaluated instead of extracts as usually used when testing biomaterials or medical devices”. Evidently, nanosized extracts may exhibit physicochemical alterations compared to the original nano-objects within the medical device, thus extracting them from a final product can lead to inaccuracies in safety assessment. Caution is warranted when applying the ISO technical report, as it employs a nanomaterial definition differing from that endorsed by the European Commission. Specifically, the ISO report considers both internal and external dimensions of the material in accordance with ISO/TS 80004-1:2015: “A material is considered a nanomaterial when it has a size at the nanoscale including external and internal dimensions, i.e., when it has a size or is composed of structures with a length of approximately between 1 nm and 100 nm” [89].

Since “nanomaterials pose specific challenges when applying test systems commonly used for medical device evaluation and when interpreting test results” [89], we can say that the advent of MDs containing nanomaterials is straining conventional safety evaluation methodologies due to the novelty of this field and persisting knowledge gaps. The cautious risk classification approach outlined in MDR 2017/745 for nanostructured MDs appears the most appropriate given the absence of normative support and numerous unresolved research issues.

## 10. Conclusions

The presence of nanostructures in medical devices promises to make available MDs with unprecedented performance dueto the distinctive properties of matter at the nanoscale. However, there is still a lack of knowledge regarding the safety and effectiveness of nanostructured medical devices. Furthermore, harmonized standards for such devices are not yet established, making it challenging to demonstrate conformity to the general safety and performance requirements outlined in the MD Regulation 2017/745.

In essence, the safety assessment of nanostructured medical devices hinges on nanoparticle release and exposure duration. It is widely recognized that the ISO 10993 series needs to be updated to address the biological impacts of nanomaterials in medical devices. In the absence of this necessary update, the following key points should be emphasized:

- Comparing test results obtained through different methodologies may be necessary to achieve the required level of certainty about safety evaluations;
- When a medical device incorporates nanomaterials, it is imperative to evaluate the latter separately from the device itself, rather than extracting them, contrary to conventional approaches used for testing biomaterials or medical devices [3];
- In general, the process of release of nanomaterials from MDs during their operational lifetime, needs to be characterized more thoroughly. Different release mechanisms may cause different modifications of the nanomaterials, with a consequent impact on their biological effect upon contact with body tissues;
- Nanoparticles of the type shed from MDs must be always tested in physiological conditions since the protein corona effect has a remarkable effect on the biological interactions of the NPs with cells/tissues [24].

## Figures and Tables

**Table 1:** Core basis of the literature evidence used in the present study.

Authors	DOI	Publication Year	Reference
van der Zee, M. et al.	10.1007/s13346-023-01359-y	2023	[25]
Li, F.Y. et al.	10.1016/j.cej.2023.143546	2023	[26]
Murugan, C. et al.	10.1016/j.jphotochem.2022.114466	2023	[27]
Lee, G. et al.	10.1016/j.addr.2022.114419	2022	[28]
Tutty, M.A. et al.	10.1007/s13346-022-01178-7	2022	[17]
Driscoll, N. et al.	10.1126/scitranslmed.abf8629	2021	[29]
Liu, H.F. et al.	10.1021/acsami.1c12352	2021	[30]
Melzer, J.E. et al.	10.1038/s41378-021-00272-z	2021	[31]
Darwish, M.S.A. et al.	10.3390/nano11051096	2021	[32]
Jesus, S. et al.	10.1021/acs.chemrestox.0c00208	2020	[33]
Sandri, G. et al.	10.1016/j.actbio.2017.05.032	2017	[34]
McCloskey, A.P. et al.	10.1002/psc.2951	2017	[35]
Naik, K. et al.	10.1166/jnn.2015.11688	2015	[36]
Bhattacharjee, S. et al.	10.2217/NNM.15.69	2015	[5]
Tran, N. et al.	10.1002/cphc.201200091	2012	[37]
Wong, P.K. et al.	10.1361/cp2007mpmd213	2008	[38]

## Data Availability

Not applicable.
